# The Longest Livers

**Published:** 1857-07

**Authors:** 


### THE LONGEST LIVERS

Are they who dwell in Palaces and Poor Houses. As contradictory, as this appears, it is not the less true. The reason of it is, in the fact, that, knowing they are provided for, the mind is at rest, and is wholly disencumbered of that eating anxiety, that care for to-morrow, which press so heavily upon the mass of mankind. The very rich and the very poor are not the healthiest; on the contrary, they are seldom entirely well; this indisposition takes away what little appetite a loafing life allows them, hence, for a short time, they eat almost nothing: this gives the stomach time to recuperate, while nature works off the surplusage, and by this double operation, they are made as well as ever in a few days. Hence, the best "Life Insurance" is to secure for yourself, at the earliest possible day, *a moderate, uniform, and* CERTAIN *income.*

